# Influence of artificial turf temperature on physical performance and muscle contractile properties in football players after a repeated-sprint ability test

**DOI:** 10.1038/s41598-020-69720-6

**Published:** 2020-07-29

**Authors:** Gabriel Calderón-Pellegrino, Leonor Gallardo, Víctor Paredes-Hernández, Jorge García-Unanue, Jesus Vicente Giménez, Enrique Colino, Jose Luis Felipe, Javier Sánchez-Sánchez

**Affiliations:** 1 Grupo IGOID, University of Castilla-La Mancha, Toledo, Spain; 2 Universidad Camilo José Cela, Madrid, Spain; 3 Faculty of Sport Sciences, Universidad Europea de Madrid, Madrid, Spain

**Keywords:** Surface patterning, Physiology

## Abstract

This study aimed to analyse the effect of playing surface temperature on muscular and thermal response to a repeated-sprint ability (RSA) test in football players. Thirty-two male football players (23 ± 5 years; 1.77 ± 0.06 m; 71.2 ± 6.7 kg) from two squads of a third-division football club participated in the study. An RSA test was carried out at a high surface temperature (45.34 ± 2.53 °C) and low surface temperature (27.21 ± 2.17 °C). Before and after this test, the muscular response of the players was assessed through tensiomyography and thermograms. The results revealed that performance in the RSA test particularly increased at a higher surface temperature, especially in the first 5 m of the 30 m sprint test. While a reduction in maximal radial displacement (Dm) in the biceps femoris post-RSA was observed at lower surface temperatures, a higher temperature on the thigh, hamstring and calf was found in the higher surface temperature group. In conclusion, higher surface temperatures had an influence on players’ thermal and tensiomyographic profile and improved performance in their repeated-sprint ability. These results suggest a need for coaches and players to be aware of these parameters to ensure adequate functionality and safety of the playing surface.

## Introduction

Football is a sport that demands maximal or near maximal actions of short duration with brief recovery periods^1^. A top-class player performs intermittent work and 150–250 brief intense actions during a game, and in terms of energy production and energy systems, high-intensity exercise periods are important^2^. Repeated-sprint ability (RSA)-based exercises are characterized by several sprints interspersed with brief recovery periods between bouts^3^. The RSA test is recognized as a valid method to reproduce performance decrement and fatigue in football players^3^. From a practical standpoint, the faster an athlete can accelerate, the greater the chance of success − for instance, evading an opponent or getting possession of the ball^4^. It has been noted that linear sprints are the most usual actions in goal situations and highlight the importance of maximal efforts and speed during decisive moments of the game^5^. Moreover, Cuadrado-Peñafiel et al.^6^ have shown that the performance of an athlete in an RSA test offers relevant information on the explosive ability of footballers, and Padulo et al.^7^ have highlighted the use of an RSA test as a tool to evaluate an athlete’s ability to recover from sprint efforts. There is a significant correlation between performance in this RSA test (3 sets of 6 × 40 m maximal shuttle sprints) and sprinting or high-intensity running distances covered during official matches in professional football players^8^. The use of the RSA protocol in previous studies has been based on the intermittent nature of football and the determinant importance of high-intensity actions during the game^2,8–10^. These significant correlations confirm that the RSA test involves the physical capacities taxed during the high-intensity phases of a match^9^. Furthermore, the authors noted that, as this test includes shuttle sprints, the muscular contractions required for decelerating and to reaccelerate body mass may potentially be beneficial in improving physiological responses and the ability to change direction, so they could be considered an appropriate training exercise for football players. The load of this kind of test can be analysed through different physiological and mechanical variables, such as body temperature^11,12^ and muscular response^13,14^.

According to the thermal profile analysis, muscle activity is one of the principal heat sources of the human body^15^. Therefore, exercise is considered to be one of the strongest influences on skin temperature (Tsk). To understand the concept, Jiang et al.^16^ briefly noted that Tsk is the result of the heat balance that is generated by the metabolism and heat loss through thermal conduction, forced and natural convection, perspiration and exhalation. It is important to note that body temperature is one of the most commonly used indicators of health status in humans and it is influenced by a tremendously large number of factors^17^. This research showed that the human thermal portrait is related to the individual fitness and expertise level, aerobic working capacity, blood lactate levels, sport specialization and sweating capacity. Moreover, a strong correlation has been found between the skin and ambient temperature. Tsk was observed to increase proportionally with the ambient temperature^18^. Additionally, Fisher et al.^19^ performed a study demonstrating that extreme environmental conditions can significantly affect Tsk. On the other hand, it has been shown that hyperthermia in the muscle temperature of players caused by hot ambient conditions markedly decreases high-intensity running at the end of a game; however, it increases peak sprinting speed^20^, and, prior to competition, maximal sprint performance may be improved when the muscle temperature is elevated^21^. Studies of the thermal response following exercise have reported both increases and decreases in Tsk immediately following exercise, depending on the type and duration of the exercise. Normally, increasing Tsk is related to constant and prolonged aerobic tasks^21,22^ , whereas studies that report decreasing Tsk primarily utilized brief intense or maximal exercises^23,24^. Malkinson^25^ affirmed that a greater intensity is related to a major increase in Tsk; however, other works have shown the opposite: there is an indirect relationship between exercise intensity and Tsk^12,26^. Nevertheless, no studies have been published regarding the effect of the RSA protocol on the thermal profile.

Muscular response, fatigue of the lower-limb muscles appears to be an important factor in elevating the risk of injury^27^. Fatigue-inducing actions include muscle stiffness, contraction speed or displacement of the muscle belly^14^ and can be determined by tensiomyography (TMG), via the application of an electrical stimulus^28^. TMG has been identified as a reliable method for analysing the risk of injury in athletes^14^ as it enables the identification of asymmetries in the lower-limb muscles and differences in muscle responses after completing fatiguing efforts such as an RSA test^29^. More specifically, this technique has been reported to have high reproducibility and reliability in measuring values like contraction time (Tc), half-relaxation time (Tr), delay time (Td), sustained contraction time (Ts) and maximal radial displacement of the muscle belly (Dm) for the medial vastus, lateral vastus, femoris rectus and biceps femoris muscles^14,27–30^.

The evolution that artificial turfs have undergone is still unable to prevent the turf from reaching higher temperatures than natural grass. This situation results in football players’ dissatisfaction, decreased performance and the possibility of causing heat-related injuries^31^. Felipe et al.^32^ and Sánchez-Sánchez et al.^33^ showed that high temperatures have a potential effect on the physiological stress of users of artificial turf. As a result, a greater compaction of the surface has been calculated when the temperature increases^34^. This aspect had an influence on mechanical properties, especially with regard to shock absorption, energy restitution and rotational resistance, making the surface harder^35^. This causes a reduction in the contact time with the surface, improving the efficiency of running technique and reducing fatigue, with longer step lengths, a more extended knee posture, and higher ankle and hip angular velocities^33,35^. These findings indicate that a change in an artificial turf’s mechanical properties can affect an athlete’s landing and acceleration mechanics^34^, thereby improving physical performance. However, negative perceptions of football players have been shown when the surface temperature is increased excessively^32^.

A large number of studies have been published about RSA in football, however few of them have analysed the effect of RSA on thermal and muscular response in football players. Moreover, the artificial turf temperature reached is considered a risk factor for players’ safety, but no studies have demonstrated the influence of temperature on players’ performance and safety. Therefore, the aim of this study was to analyse the effect of playing surface temperature on muscular and thermal response to a repeated-sprint ability test in football players.

## Results

### RSA test

**Figure 1:**
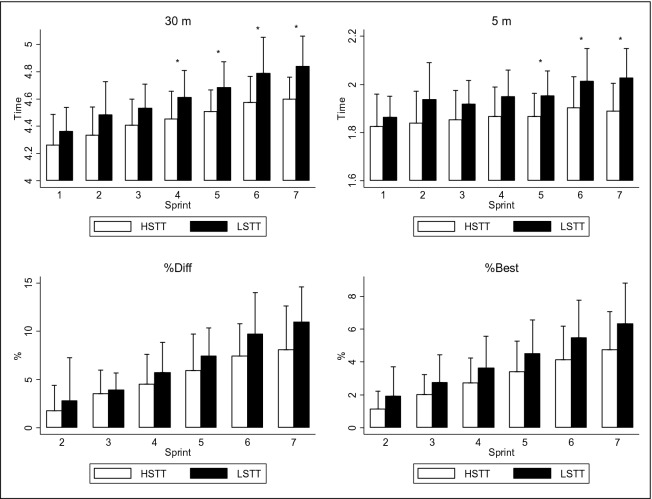
30 m (**A**) and 5 m (**B**) time and performance deterioration profile; RSA_DEC_ (**C**) and RSA_CHANGE_ (**D**) for the RSA test (7 × 30 m). RSA_CHANGE_: ((worst time – best time)/best time) * 100). RSA_DEC_: ((total sprint time − best time * 7)/best time * 7) * 100). HSTT: high surface temperature test. LSTT: low surface temperature test. *p < 0.05; significant differences between high (white) and low (black) surface temperature (n = 32). Data are presented as mean and SD.

Analysis of the RSA test revealed significant differences in time and accumulated fatigue in relation to the surface temperature (*p* < 0.05; Fig. 1). Players revealed worse times in the RSA test when the surface temperature was lower, especially in the last sprint, in 5 m (+ 0.14 s; CI 95%: 0.05–0.23; ES: 1.15; *p* = 0.005) and 30 m (+ 0.24 s; CI 95% 0.09–0.39; ES: 1.25; *p* = 0.003). The difference in performance deterioration between the last and the first sprint was greater when the surface temperature was lower in RSA_DEC_ (+ 4.42%; CI 95% 3.53–5.30; ES: 2.07; *p* < 0.001) and RSA_CHANGE_ (+ 8.14%; CI 95% 5.87–10.40; ES: 2.00; *p* < 0.001).

### Thermal analysis

Differences in different muscle temperatures after the RSA test between groups (*p* < 0.05) were revealed (Table 1). Players showed a higher temperature on the thigh (+ 0.8 °C; CI 95% 0.2–1.3; ES: 1.06; *p* = 0.006), hamstring (+ 1.1 °C; CI 95% 0.6–1.6; ES: 1.63; *p* < 0.001) and calf (+ 1.0 °C; CI 95% 0.6–1.4; ES: 1.70; *p* < 0.001;) at a higher surface temperature; however, no significant differences were found between groups before the RSA test (*p* > 0.05). The contractile muscle properties of the players were independent of the surface temperature (p > 0.05). However, the players showed a significant reduction in Td (− 1.18 ms; CI 95% − 2.27 to − 0.10; ES: 0.50; *p* = 0.034) and Dm (− 2.62 mm; CI 95% − 4.02 to − 1.21; ES: 0.85; *p* = 0.001) in the biceps femoris at a higher surface temperature. Similarly, a reduction of the Dm in the BF after the RSA test was identified when the surface temperature was lower (− 1.37 mm; CI 95% − 2.70 to − 0.05; ES: 0.50; *p* = 0.042).

**Table 1:** Results of the tensiomyography and thermography before and after the RSA test (7 × 30 m) according to the temperature of the surface.

Variable	Temperature	Pre	Post
**Thermography**			
Thigh (°C)	HSTT	34.3 ± 0.6	33.9 ± 0.6*
	LSTT	34.1 ± 0.9#	33.2 ± 0.8
Hamstring (°C)	HSTT	34.3 ± 0.5	34.1 ± 0.4*
	LSTT	34.0 ± 0.9#	33.0 ± 0.9
Calf (°C)	HSTT	34.2 ± 0.6#	33.7 ± 0.5*
	LSTT	33.9 ± 0.7#	32.7 ± 0.7
**Tensiomiography**			
*BF*			
Td (ms)	HSTT	24.83 ± 2.57#	23.65 ± 2.21
	LSTT	24.57 ± 1.60	25.08 ± 2.12
Tc (ms)	HSTT	39.42 ± 17.18	33.18 ± 12.04
	LSTT	41.82 ± 18.40	41.60 ± 15.07
Dm (mm)	HSTT	9.99 ± 3.35#	7.37 ± 2.83
	LSTT	7.94 ± 3.09#	6.56 ± 2.43
*RF*			
Td (ms)	HSTT	23.55 ± 1.81	23.30 ± 2.00
	LSTT	23.34 ± 1.98	23.55 ± 2.04
Tc (ms)	HSTT	27.20 ± 4.20	26.29 ± 2.63
	LSTT	26.47 ± 3.06	27.16 ± 1.87
Dm (mm)	HSTT	11.84 ± 3.56	10.79 ± 3.29
	LSTT	9.09 ± 2.24	9.74 ± 1.95

*Significant differences between the high surface temperature test (HSTT) group and low surface temperature test (LSTT) group; # significant differences between pre and post; BF, biceps femoris; RF, rectus femoris; Td, delay time; Tc, contraction time; Dm, muscle radial displacement.

## Discussion

This study analysed the effect of surface temperature on thermal and muscular response before and after an RSA test in football players. The results revealed that performance in the RSA test increased at a higher surface temperature (45.34 ± 2.53 °C), especially in the 5 m and 30 m of the sprint. On the other hand, significant differences post-RSA were found in players’ thermal profiles, depending on the field temperature. In regard to contractile properties, the RSA test decreased Dm in the BF in both groups (HSTT and LSTT) and Td decreased only in the HSTT post-RSA. However, no significant differences were found regarding field temperature.

In this study, players improved their performance in the RSA protocol at higher field temperatures, probably due to the shock absorption (SA), which is lower when the temperature is high; consequently, the surface becomes harder and the subject is able to run faster^33^. It is interesting to note that shock absorption is the ability of a surface to reduce the impact force of a load. Contrarily, if the temperature is lower, the SA is higher, thus running efficiency and speed decrease, and consequently a higher level of fatigue will be found^35^. Similar results were found in Sánchez-Sánchez et al.^33^, who noted that users’ performance can be improved if the surface becomes too hard because of the lower contact time during running and the less frequent reuse of the stored elastic energy of the surface with a higher damping capacity. Moreover, it has been found that too hard fields^36^ and too soft fields^35^ can adversely affect sport performance and increase the risk of injury. Thus, environmental conditions have an influence on the mechanical behaviour of artificial turf, and it has been proven that temperature alters the mechanical properties of the surface. However, other studies found that temperature, in contrast to intensity of use, did not significantly alter the mechanical behaviour of a surface, although Sánchez-Sánchez et al.^33^ reported a lower SA at a higher surface temperature.

Another element of the present study was muscular response analysed through tensiomyography (TMG), which has been identified as a reliable method for detecting differences in muscle responses after completing fatiguing efforts such as an RSA test^29^. Delay time (Td), radial displacement (Dm) and contraction time (Tc) in the biceps femoris (BF) and rectus femoris (RF) have been measured at high and low surface temperatures before and after the RSA test. Our study found a decrement in Dm in the BF after the RSA protocol, mostly at high surface temperatures. These results are in line with other investigations that reported a decrease in Dm after high-intensity exercises^29^. All these studies used variations of high-intensity resistance or endurance training, or interval training, over short periods of time. This reduction is interpreted as an increase in muscle stiffness that occurs after high-intensity efforts^37^. Furthermore, measurements of Dm were lower following high-load resistance exercise than after workload-matched high-volume training^38^, and Wiewelhove et al.^39^ reported that Dm tended to be reduced following six days of high-intensity interval training. In our study, the RSA test caused a similar reduction in Dm. Contrary to our findings, two other studies revealed an increase in Dm after ultra-endurance exercise using TMG, namely an Ironman triathlon^40^ and an uphill marathon^39^, whilst others showed no change^41^. Thus, the type of exercise can determine the contractile properties of the muscle, reducing Dm after high-intensity and brief efforts and increasing it after longer and less intense exercises^13^. According to the rectus femoris analysed in our study, no significant changes were found in any contractile property of the muscle in the RSA test at high and low temperatures, probably due to the small activation of this muscle in high-intensity efforts like the RSA, in contrast to the BF.

As for Tc, no significant differences were found in our investigation before or after the RSA test in the low and high surface temperature groups. However, conflicting results were reported for Tc, which showed a post-fatigue increase^40^ or decrease^39^, or no change^29^.

According to the physiological load, no significant differences were found in the HR variability parameter between high and low temperature groups during the RSA test. Similarly to studies by Hughes et al.^42^ and Sánchez-Sánchez et al.^33^, there was no influence of the surface on a player’s internal load, although physical performance was different according to the surface temperature. In conclusion, surface temperature had an influence on the players’ thermal and tensiomyographic profile and on their repeated-sprint ability test performance. This study increases knowledge about the effect of an RSA test on the thermal and tensiomiography responses of football players because no studies have been published about this topic so its application in this area could be useful. This study contributes towards an adequate use of artificial turf with the objective of converting it into a safe surface that ensures a good performance for football players.

## Methods

### Subjects

A group of 32 male football players (23 ± 5 years; 1.77 ± 0.06 m; 71.2 ± 6.7 kg) participated in the study. The subjects were part of two squads of a third-division football club, and they all provided written consent before participating in the research, in accordance with the Declaration of Helsinki. Test procedures and possible risks were explained in detail in the written consent. Two test sessions were performed across two different days. Each session included two tests: high temperature surface test (HSTT, at 16:00) and low surface temperature test (LSTT, at 20:00). Subjects were randomly distributed into two groups. The first group performed the first test in HSTT (day 1) and afterward in LSTT (day 2). The second group performed the first test in LSTT (day 1) and afterward in HSTT (day 2). All participants were healthy and not injured, and they were required not to perform any exhausting activity at least 24 h before testing.

### Procedures

The data collection process took place immediately after the end of the regular season. The experimental tests were carried out during four different sessions distributed over two consecutive days, two sessions each day. Sessions 1 and 3 took place at 16:00 h, and sessions 2 and 4 at 20:00 h. The main part of the test protocol consisted of a repeated-sprint ability (RSA) test carried out at ambient temperature. The mean surface temperature during the tests carried out at 16:00 and 20:00 was 45.34 ± 2.53 °C and 27.21 ± 2.17 °C, being categorized as high surface temperature tests (HSTTs) and low surface temperature tests (LSTTs), respectively. The ambient temperature oscillated between 30 and 35 °C. All temperature measures were performed using a PCE-T 318 (PCE Holding GmbH, Meschede, Germany). Before and after the RSA test, the muscular response of the players was assessed by means of tensiomyography (TMG-100 System electrostimulator, TMG-BMC d.o.o., Ljubljana, Slovenia), and digital thermal images (thermograms) were recorded for the front and rear surfaces of the lower limb by means of a FLIR T420bx infrared thermographic camera (FLIR Systems Inc., Wilsonville, OR, USA). Players’ heart rate (HR) was continuously monitored during the RSA test by means of an HR monitoring band (Firstbeat Technologies Ltd., Finland). The ambient temperature and surface temperature at the time of performing the RSA test were recorded.

### Tensiomyography (TMG)

**Figure 2:**
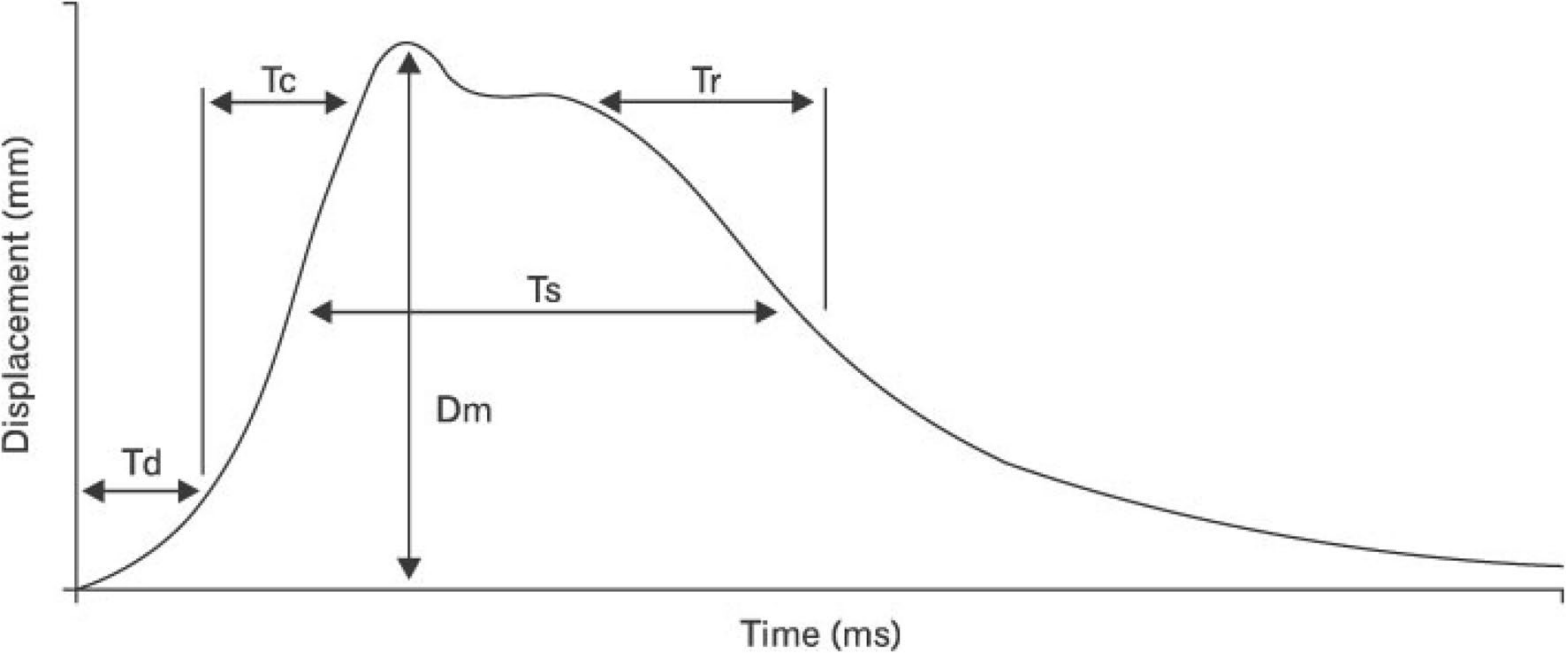
TMG parameters definition.

The muscular response and the lateral symmetry of both the rectus femoris (RF) and biceps femoris (BF) were assessed. The contraction time (Tc), delay time (Td) and maximal radial displacement of the muscle belly (Dm) were recorded under basal conditions and after the RSA test. Figure 2 represents the meaning of those variables during a typical muscle contraction. The stimulus was of 1 ms, giving four stimuli to each muscle, varying the amplitude (25, 50, 75 and 100 mAp). The RF was measured with the subject in a supine position with a knee flexion of 120° with the help of a triangular foam cushion. The BF was measured with the subject in a prone position and the knee flexed 5º with the help of a foam cushion^43^.

Dm (mm) is a parameter that reflects the maximal radial displacement of the muscle belly as a consequence of muscle contraction and depends on the flexibility and tone of the muscular tissue. Therefore, Dm values increase when the explosive force is developed, involving high movement amplitude, and decrease under the conditions of a high muscular tone. Td (ms) is the time lapse between the transmission of the electric stimulus and the onset of muscle contraction (10% Dm). Tc (ms) is the time between the moment when the muscular contraction is 10% of the Dm and the moment when the contraction reaches 90% of the Dm.

The response to the electric stimulus was measured by placing a digital Dc-Dc transducer Trans-Tek (GK 40, Panoptik d.o.o., Ljubljana, Slovenia) perpendicular to the muscle belly, along with two self-adhesive electrodes (TMG electrodes, TMG-BMC d.o.o. Ljubljana, Slovenia) placed equidistantly at a distance of 50–60 mm from the digital transducer. Sensor and electrode positions were marked with a permanent marker to ensure that all measurements were performed at the same point^44,45^. All measurements were carried out by the same expert technician.

### Thermograms

Data collection followed the standards proposed by the European Association of Thermology^44^. Thermograms were assessed before and after the RSA tests in an air-conditioned room, registering skin temperatures to a sensitivity of < 0.1 °C. All the thermograms were taken in similar conditions: the room temperature was maintained at 23 ± 0.3 °C, air humidity ranged between 35 and 45%, and the distance between the subject and the camera was always 3 m. Subjects began and finished the RSA test one after the other, so the time duration between exercise and testing time was the same for each participant. Tests were preceded by a 10-min acclimatization period for all subjects, surpassing the minimum time of 8 min of stabilization proposed by Roy et al.^46^. For each participant and session, two series of thermograms were performed on the front and rear surfaces of the lower limbs (lower legs and thighs) in a standing position. The first was taken 15 min before the RSA test, and the second 15 min after. Prior to and during the procedure, the subjects were asked to avoid any sudden and intense movement, or rubbing, scratching or crossing their legs. During the tests, subjects were dressed in shorts and sport shoes, so the selected skin surface areas were continuously exposed during the exercises and the measurements. Body regions of interest (ROIs) analysed included the thighs, hamstring and calf. Computerized image analysis allowed selection of the measurement area on the thermograms. These areas were selected by a rectangle bounded by the software Flir Tools (FLIR Systems Inc., Wilsonville, OR, USA), which provided the average temperature (Tmean) from each analysed ROI. The index of human skin emissivity was assumed to be 0.98^47^.

### Repeated-sprint ability test (RSA)

The RSA test included seven repeated sprints of 30 m, with 20 s of active recovery between each sprint. Four pairs of photocells (Witty, Microgate, Bolzano, Italy) placed at 0, 5 and 30 m were used to assess performance in this test. This test was performed according to the methodology proposed in previous studies^48^. The best sprint time (RSA_BEST_), the mean time (RSA_MEAN_), the total time (RSA_TT_), the per cent sprint decrement RSA_DEC_ ((7-sprints total time − best time * 7)/best time * 7) * 100) and the per cent difference between best and worst sprints during the RSA test (RSA_CHANGE_ ((worst time – best time)/best time) * 100) were also calculated^48^. Before the RSA, participants carried out a standardized warm-up consisting of 5 min of running, 5 min of joint mobility and three bouts of 30 m sprints of increasing intensity. The warm-up concluded with two 30 m sprints at maximum intensity with 4 min of active recovery. These two previous sprints were used as a control measure to ensure that players would perform the RSA test at maximal intensity. During this test, if the time of the first sprint was higher (> 5%) than the best individual sprint performed prior to the beginning of the test, the RSA test was not considered valid and the player had to repeat the test after 5 min of recovery^34^.

### Statistical analyses

SPSS 21.0 (IBM Corp, New York, USA) was used for the data analysis. A descriptive analysis (mean ± SD) of the TMG test results, the temperatures of the analysed body surfaces and the performance parameters of the RSA test was performed. Kolmogorov–Smirnov analyses indicated all data were normally distributed; therefore, parametric tests were used. Two groups were separated according to the surface temperature, using a K-means analysis. The high and low temperature groups had a surface temperature of 45.34 ± 2.53 °C and 27.21 ± 2.17 °C, respectively. Two-way analysis of variance (ANOVA) was used to analyse the differences in the TMG and the thermographic variables regarding the time (before and after the exercise) and temperature (HSTT and LSTT). Differences in performance and between the first and last sprint in the RSA test at both temperatures were analysed using a two-way ANOVA. A Bonferroni post hoc test was employed to study pairwise differences. The confidence interval and the effect size (ES; Cohen’s d) of the pre- to post-differences for all variables (CI of 95%) were calculated. The ES was evaluated with the following criteria: 0 to 0.2 = trivial; 0.2 to 0.5 = small; 0.5 to 0.8 = moderate; and > 0.8 = large^49^. The level of significance was established at *p* < 0.05.

### Ethical statement

The study protocol was approved by the Local Ethics Committee (Toledo Hospital; CEIC61). All research was performed in accordance with the Code of Ethics of the World Medical Association (Declaration of Helsinki) and all of the participants signed an informed consent form in which the test procedures and possible risks were explained.
